# Designing and Developing Interprofessional Learning Experiences in Palliative Care: A Collaborative Workshop Approach

**DOI:** 10.1089/pmr.2024.0081

**Published:** 2025-04-16

**Authors:** Carolyn Kezar, Justine McGiboney, Michael D. Barnett, Richard Taylor, Rebecca Edwards, Ella H. Bowman, Elizabeth McAlister, Moneka A. Thompson, Tara Schapmire, Chao-Hui Sylvia Huang

**Affiliations:** ^1^ Division of Gerontology, Geriatrics and Palliative Care, Department of Medicine, University of Alabama at Birmingham, Birmingham, Alabama, USA.; ^2^ Department of Veterans Affairs, Birmingham, Alabama, USA.; ^3^ Department of Emergency Medicine, University of Alabama at Birmingham, Birmingham, Alabama, USA.; ^4^ School of Nursing, University of Alabama at Birmingham, Birmingham, Alabama, USA.; ^5^ Division of General Internal Medicine and Geriatrics, Oregon Health and Science University, Portland, Oregon, USA.; ^6^ Division of Palliative Medicine, Department of Medicine, University of Louisville School of Medicine, Louisville, Kentucky, USA.; ^7^ O’Neal Comprehensive Cancer Center, University of Alabama at Birmingham, Birmingham, Alabama, USA.

**Keywords:** collaborative learning, competency-based education, interdisciplinary training, interprofessional education, palliative care

## Abstract

**Background:**

Team-based care is vital in palliative care, but there is limited interprofessional education (IPE) among health care providers, leading to siloed learning. This project addresses this gap by developing a workshop focused on the active dying process, promoting shared competencies among palliative medicine, geriatrics, nursing, and psychology subspecialty learners.

**Objective:**

We aimed to design, implement, and evaluate an Interprofessional Education Exchange (IPEX) Death and Dying workshop to foster interdisciplinary collaboration and improve participants’ comfort with palliative care competencies.

**Design:**

A full-day, case-based workshop was developed using the Analysis, Design, Development, Implementation, Evaluation model, emphasizing experiential and collaborative learning.

**Setting/Subjects:**

Two workshops were held at a tertiary southeastern academic university in the United States (US) in 2022 (cohort 1) and 2023 (cohort 2). A total of 25 participants, including palliative medicine fellows, geriatric medicine fellows, advanced nurse practitioner students, and psychology residents, completed the workshops.

**Measurements:**

Participants’ comfort with palliative care competencies, perceptions of interprofessional collaboration, and qualitative feedback were assessed using post-workshop surveys.

**Results:**

Participants’ comfort in providing anticipatory guidance, addressing spiritual distress, and supporting grief and bereavement improved. Interprofessional collaboration and professional growth, particularly in communication and understanding each other’s roles and responsibilities in caring for the actively dying, also increased.

**Conclusions:**

The collaborative IPEX Death and Dying workshop has been shown to enhance competencies and foster interprofessional collaboration among palliative care subspecialty learners across four disciplines. This model holds potential for broader implementation across health care settings to improve care for the seriously ill patients.

## Background

Teamwork is vital to the ethos of palliative care (PC), where professionals from diverse disciplines, such as physicians, nurses, social workers, and chaplains, collaborate to leverage their unique expertise to provide holistic patient care.^1^ However, health care providers across multiple specialties often lack the foundational skills necessary to deliver effective PC for the seriously ill.^2^ Moreover, many subspecialty training programs operate in professional silos, often within small cohorts of learners and educators, highlighting the pressing need to synergize teaching efforts across disciplines. We expect hospice and palliative care (HAPC) clinicians to work together effectively, yet we often do so without providing them with training opportunities to understand each discipline’s unique professional roles and expertise, or how to best learn from one another. Collaborative interprofessional education (IPE) can address these gaps in knowledge and ultimately improve care outcomes for patients with serious illness.^3^

IPE in PC is an emerging approach, supported by a growing body of literature.^4–7^ This model emphasizes collaborative learning across multiple disciplines, fostering teamwork and communication that are essential for delivering high-quality PC. A 2023 systematic review identified 39 interprofessional PC educational studies, the majority of which focused on early-stage medical and nursing learners, with less emphasis on subspecialty learners, such as fellows and residents, who will soon require these skills and knowledge to effectively integrate into the interprofessional PC workforce.^8–9^

One significant challenge in designing interprofessional learning experiences is understanding the varying expertise of different attendee groups and creating an experience that leverages shared competencies to foster collaborative learning. While much of the work in IPE has focused on early learners, case-based learning (CBL) has been effectively utilized to elicit diverse perspectives and insights among multidisciplinary participants.^10^

To further the effort to incorporate interprofessional learning later in the training experience, the authors have previously identified an intersecting set of competencies among PC, geriatrics, nursing, and psychology learners on which to center learning experiences. Competencies related to managing and meeting the psychosocial, cultural, and spiritual care needs of the dying had the highest percent match among disciples, followed by rapport building and shared decision-making, and patient–provider communication.^11^

## Objective

This study aims to address the gap in interprofessional PC education by developing, implementing, and evaluating a novel workshop on the active dying process, focusing on shared competencies among palliative medicine fellows, geriatrics fellows, advanced nursing students, and psychology residents, while detailing the collaborative design process and assessing educational outcomes.

## Learning Design and Development

The Analysis, Design, Development, Implementation, Evaluation (ADDIE) model was instrumental in developing the learning experience.^12^ This curriculum design framework guides each phase of the process, with tangible outputs from each step informing the next. This approach allows for iterative feedback as the design unfolds. By following the ADDIE model, we implemented a systematic approach to creating an effective IPE workshop.

Our team participated in the University of Louisville’s NCI R25 grant funded Interprofessional Education Exchange (IPEX) program aimed at improving cancer care.^6,13^ This experience provided valuable insights that directly supported our design and development process for educational initiatives.

### Needs assessment

The needs assessment phase was driven by a comprehensive literature review of learner needs and preferences in IPE, with an emphasis on competency-based training in PC. Given that our academic medical center has established subspecialty training programs in HAPC, we conducted needs assessment to determine the content focus of the IPE curriculum. Foundational curricular used for needs assessment included Hospice and Palliative Medicine Milestones, Geriatric Physician Fellowship Curricular Milestones, Hospice and Palliative Advanced Practice Nurse Competencies, and Palliative Care Competencies for Psychologists.^14–17^

**FIG. 1.**
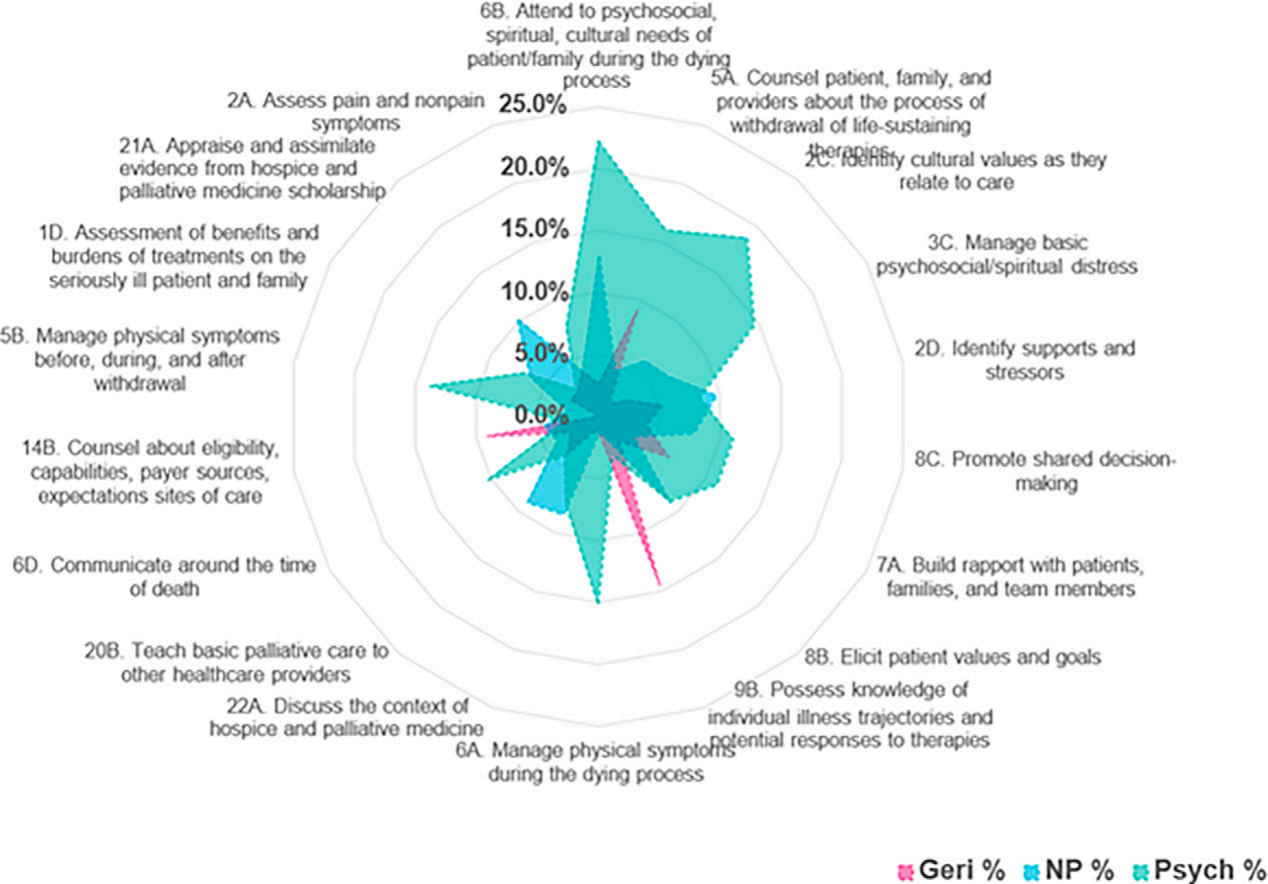
Findings from the Palliative-Geriatrics-Nursing-Psychology interprofessional education (IPE) curriculum cross-match identify an intersecting set of competencies across all four disciplines. Total match percentages highlight overlapping competency areas and discipline-specific competencies. Geri, geriatric medicine; NP, nurse practitioner; Psych, psychology. Total match % highlight shared/overlapped competency areas and discipline-specific competencies.

Informed by the evaluation of existing competencies and cross-matching between each program’s subspecialties (PC, geriatrics, nursing, and psychology), we conducted five cycles of cross-disciplinary structured review using 1:1 discipline assessment dyads. These dyads paired clinician educators from complementary specialties: palliative-geriatrics, geriatrics-nurse practitioner (NP), NP-psychology, and psychology-palliative. A final independent review by a program coordinator and instructional designer was conducted to ensure alignment. A consensus on IPE core training competencies was achieved through expert discussion and analysis. Findings from the Palliative-Geriatrics-Nursing-Psychology IPE curriculum cross-match identify an intersecting set of competencies across all four disciplines (see Fig. 1).^11^

### Design

Once consensus was reached regarding shared competencies, we identified areas of greatest overlap that matched both our team’s educational expertise and the needs of our program’s current curricula. The decision was made to focus on the active dying process due to its universal relevance across disciplines. Learning objectives were categorized by core concepts: communication; care options and resources; cultural, religious, and spiritual care; grief and family support; and symptom management. These objectives were further grouped by Bloom’s Taxonomy category: knowledge, skills, and attitudes, ensuring a balanced approach to learning outcomes. They were also aligned with the National Consensus Project domains and Interprofessional Education Collaborative competencies.^18–19^ Based on these factors, the workshop was structured around pre-death, peri-death, and post-death phases.

### Development

The development phase focused on creating a full-day workshop to establish an immersive and collaborative learning environment. Given that learners had minimal prior interactions and came from different discipline-specific training experiences, we recognized the importance of face-to-face engagement to foster trust and community both among learners and faculty. The emotional and complex nature of death required a psychologically safe and supportive environment. Social activities, such as breakfast, ice breakers, and informal discussions, were used to cultivate community and trust throughout the day. This was critical in the design of both the physical and social learning space.

Building on the structured approach provided by the ADDIE model, we applied adult learning theories to ensure our educational design fostered active engagement and deep learning through collaboration. The development of collaborative learning activities was informed by several educational theories including Vygotsky’s Social Constructivism, Dewey’s Experiential Learning Theory, and Papert’s Constructionist principles.^20–22^ These theories underpin the use of collaborative techniques such as group discussions, role-playing, and CBL activities to encourage active engagement.^23^

### Implementation

The IPEX Death and Dying workshop was implemented as a full-day, intensive interprofessional learning experience, designed to immerse participants in collaborative PC practice, where shared norms, values, and skills in the care of the dying could be developed and refined.

The scheduling of the workshop took into account the semester calendar of remote learning advanced practice nursing students, aligning with their on-campus availability in the late fall. This also allowed time for relationship-building within specialty groups after summer start dates, fostering psychological safety and trust before the workshop.

### Workshop structure

Workshop sessions were organized into three key phases—pre-death, peri-death, and post-death—each designed to guide learners through the continuum of care for dying patients. These phases provided a structured framework for integrating interdisciplinary collaboration into each aspect of the dying process.

The pre-death session focused on anticipatory guidance, psychological experiences, and physical symptoms preceding death. After a brief didactic session, participants engaged in a timeline activity where they collaboratively identified common symptoms and experiences in the dying process, followed by small and large group discussions. An additional activity focused on the shared construction of a care setting matrix, describing the resources, care providers, impacts, and burdens of different care settings for patients and caregivers at the end of life and how that impacts symptom management and psychosocial support.

The peri-death session organized learners in discipline-specific groups to create presentations or using role-play that demonstrated their respective roles during a patient’s death (e.g., death pronouncement, support of the family/caregivers, and immediate postmortem care of the body). This work was guided by the question: what is important for others to know about what you are doing around the time of death? Workshop faculty supported all small group processes including preparation, presentations, and post-presentation group discussion and feedback.

Lastly, the post-death session addressed family grief and bereavement support. First, learners participated in a didactic session followed by discussion on best communication practices in times of grief. Each participant then drafted a bereavement card to a family member of a patient they had cared for. This activity fostered personal reflection and emotional engagement with the material.

### Setting/subjects

Two IPEX Death and Dying workshops were conducted in a collaborative learning classroom setting at a southeastern tertiary academic university in the US in 2022 (cohort 1) and 2023 (cohort 2). A total of 25 participants comprised of Hospice and Palliative Medicine fellows (*n* = 8), Geriatric Medicine fellows (*n* = 1), advanced practice nursing students (*n* = 9), as well as palliative psychology learners (*n* = 7). The workshops were facilitated by an interprofessional faculty team including PC physicians, geriatric physician, graduate-level nurse faculty, a psychologist, a chaplain, and instructional design specialist.

### Measurement and evaluation

The multifaceted evaluation strategy was developed during the design process and was informed by both the learning objectives and Kirkpatrick’s Four-Level Training Evaluation Model, a framework widely recognized in medical education for assessing educational interventions. As this represented the first iterations of the IPEX Death and Dying workshop, our primary evaluation focus centered on understanding learners’ immediate reactions to the interprofessional learning experience, assessing perceived changes in competency across different professional roles, identifying areas for program improvement, and gathering preliminary evidence to support broader implementation of the workshop model. Our approach focused primarily on Level 1 (Reaction) and Level 2 (Learning) of Kirkpatrick’s framework, examining both participants’ immediate responses and their self-reported learning outcomes through a post-workshop evaluation during the final reflection and debrief session. This 10-minute survey incorporated three components: (1) core competency assessment to measure learning, (2) perceptions of interprofessional collaboration to gauge reactions to the interprofessional format, and (3) qualitative feedback on the overall experience to capture both reaction and learning dimensions.

A retrospective pre- and post-workshop measure assessed participants’ comfort levels regarding key PC competencies identified from our previous work on curriculum cross-match.^11^ These competencies included providing anticipatory guidance about the dying process, assessing family dynamics and coping mechanisms, addressing existential and spiritual distress, participating in death pronouncement and postmortem care, and supporting families through grief and bereavement. Participants rated their comfort using a 4-point Likert scale, with topics reflecting the major themes of the workshop.

Student Perceptions of Interprofessional Clinical Education-Revised Instrument, Version 2 (SPICE-R2)^24^ was used to assess perceptions of interprofessional collaboration of workshop participants. The SPICE-R2 is widely used in the IPE literature, which includes items such as, “Working with students from different disciplines enhances my education,” and “Patient/client satisfaction is improved when care is delivered by an interprofessional team.”

Additionally, qualitative feedback was gathered through open-ended questions designed to allow participants to reflect on the most impactful aspects of the workshop and provide suggestions for improvement.

Descriptive analysis was conducted using SPSS 28.0 to summarize the demographic characteristics of workshop participants and the retrospective post-workshop evaluations. Thematic analysis was performed to identify major themes and subthemes that emerged from the qualitative learner feedback.^25^ These evaluation strategies enabled both quantitative and qualitative analyses, offering a comprehensive understanding of the workshop’s effectiveness.

## Results

### Demographics

The IPEX workshops have included 25 total participants to date, with cohort 1 (*n* = 11) and cohort 2 (*n* = 14). The average age was 36 years, with over half in the 25 to 34 age range. Over half (60%) of the participants were female. Ethnicity data were collected with 72% of participants identifying as White, 16% as Black or African American, and 12% as Asian. Regarding professional background, 32% were palliative medicine fellows, 36% were from a nursing background, 28% from palliative psychology, and 4% geriatric medicine. Nearly 50% reported having never or rarely participated in IPE prior this workshop (see Table 1).

**Table 1.** Demographic Characteristics of IPEX Death and Dying Workshop Participants (*n* = 25)

Characteristics	Total (*n* = 25)		Cohort 1 (*n* = 11)		Cohort 2 (*n* = 14)	
	*n*	%	*n*	%	*n*	%
Age in years (M, [SD])	35.64 [8.92]		33.91 [7.05]		37.00 [10.21]	
25–34	15	60	7	63.6	8	57.1
35–44	5	20	3	27.3	2	14.3
≥45	5	20	1	9.1	4	28.6
Gender						
Females	15	60	6	54.5	9	64.3
Males	9	36	5	45.5	4	28.6
Preferred not to answer	1	4	11	0	1	7.1
Ethnicity						
White	18	72	10	90.9	8	57.1
Black or African American	4	16	1	9.1	3	21.4
Asian	3	12	0	0	3	21.4
Disciplines						
Palliative Medicine	8	32	4	36.4	4	28.6
Geriatric Medicine	2	8	1	9	1	7.1
Nursing	8	32	3	27.3	5	35.7
Palliative Psychology	7	28	3	27.3	4	28.6
Frequency of IPE learning						
Never	3	12	0	0	3	21.4
Rarely	9	36	7	63.6	2	14.3
Sometimes	8	32	3	27.3	5	35.7
Often	5	20	1	9.1	4	28.6

IPE, interprofessional education; IPEX, Interprofessional Education Exchange.

### IPE core competencies

A comparison survey of participant comfort levels before and after the IPEX workshop across the five domains shows distinct improvements in each area. First anticipatory guidance, before the workshop 40% of participants reported low comfort in discussing future care needs with patients and families. Post-workshop scores showed improvement, with 85% of participants expressing increased comfort, indicating a positive change in their ability to provide anticipatory guidance. Next, comfort in navigating family dynamics and supporting coping mechanisms saw substantial gains. Prior to the workshop, only one-third (33%) of participants felt confident addressing complex family relationships and coping strategies. Following the workshop this percentage rose to 80%, representing a 47 percentage point increase in comfort levels. Regarding existential and spiritual distress, only 25% of participants reported feeling comfortable managing existential or spiritual distress initially. Following the workshop, this figure increased to 75% marking one of the largest improvements across all domains, with a 50 percentage point rise in comfort levels. Comfort levels in handling death pronouncement and postmortem care also improved notably. Prior to the training, only 30% of participants felt at ease with these tasks, while post-workshop results showed that 70% of participants were now comfortable. Lastly, participants also showed considerable growth in their ability to provide grief and bereavement support. Before the workshop, just 30% of participants felt confident in this area. After the workshop, 78% reported comfort with a 48 percentage point increase.

### Perceptions of interprofessional clinical education

The SPICE-R2 outcomes indicate positive shifts in perceptions of interprofessional teamwork and collaboration among the workshop participants (see Table 2). Nearly all participants (96%) agreed that working with students from different disciplines enhanced their education and ability to work effectively on an interprofessional team. Additionally, 100% of learners felt their role within the interprofessional team was clearly defined, and 92% now understood the roles of other health professionals. In terms of patient outcomes, 100% of participants agreed that patient satisfaction and patient-centeredness improved with care delivery by an interprofessional team, though fewer (76%) believed it contributed to reduced health care costs.

**Table 2.** Student Perceptions of Physician-Pharmacist Interprofessional Clinical Education Instrument, Version 2 (SPICE-2) Outcomes

Factors	Items	*N* (%)		
		Disagree/strongly disagree	Neutral	Agree/strongly agree
Interprofessional teamwork and team-based practice	1. Working with students from different disciplines enhances my education	0 (0)	1 (4)	24 (96)
	4. Participating in educational experiences with students from different disciplines enhances my ability to work on an interprofessional team	0 (0)	0 (0)	25 (100)
	7. Health professional students from different disciplines should be educated to establish collaborative relationships with one another	0 (0)	1 (4)	24 (96)
	10. During their education, health professional students should be involved in teamwork with students from different disciplines in order to understand their respective roles	0 (0)	1 (4)	24 (96)
Roles/responsibilities for collaborative practice	2. My role within an interprofessional team is clearly defined	0 (0)	0 (0)	25 (100)
	5. I have an understanding of the courses taken by, and training requirements of other health professionals	2 (8)	4 (16)	19 (76)
	8. I understand the roles of other health professionals within an interprofessional team	0 (0)	2 (8)	23 (92)
Patient outcomes from collaborative practice	3. Patient/client satisfaction is improved when care is delivered by an interprofessional team	0 (0)	0 (0)	25 (100)
	6. Health care costs are reduced when patients/clients are treated by an interprofessional team	2 (8)	6 (24)	19 (76)
	9. Patient/client-centeredness increases when care is delivered by an interprofessional team	0 (0)	0 (0)	25 (100)

### Learner feedback

Table 3 summarizes major themes and subthemes that emerged from the thematic analysis of learner feedback from the IPEX Death and Dying workshop. Findings revealed several key areas of personal growth and professional impact. Participants reported professional growth with many citing improved communication skills that would likely enhance patient and family quality of care. One participant stated “*The patient/family likely will receive higher quality of care because I learned better communication.*” Others highlighted the development of empathy, expressing a deeper connection to the emotional and spiritual needs of patients and families. Additionally, participants gained self-awareness and role clarity, with one remarking “*This workshop helped clarify my role in the interdisciplinary team, making me more effective*.” Additionally, learners described improved understanding of other team members’ skills and how to leverage others’ abilities to better meet patient care needs.

**Table 3.** Thematic Analysis of Workshop Feedback

Domain	Major themes	Subthemes	Selected quotes
Personal and professional impact	Professional growth	Improved skills in communication and patient care	“The patient/family likely will receive higher quality of care because I learned better communication.”
	Enhanced communication	Better ways to interact with patients/families	“I learned better ways to communicate effectively with both patients and their families.”
	Empathy development	Attuned to emotional needs of patients	“I now feel more attuned to the emotional and existential needs of my patients.”
Learning about self and team role	Self-awareness	Greater understanding of individual roles	“I realized the importance of understanding my unique contributions within the team.”
	Role clarity	Clearer understanding of interdisciplinary responsibilities	“This workshop helped clarify my role in the interdisciplinary team, making me more effective.”
	Team integration	Increased appreciation for teamwork	“I have a new appreciation for the roles my colleagues play in patient care.”
Workshop improvement suggestions	Group collaboration	Desire for more interactive peer-learning	“More group collaborative exercises would make the workshop even better.”
	Duration of sessions	Preference for shorter, more focused sessions	“The workshop could be shorter for more focus and better retention of content.”
	Future workshop content	More case studies or practical exercises requested	“More case-based studies would be helpful to apply the learned concepts in practice.”
Best aspects of workshop	Engaging presentations	Relatable and emotionally impactful speakers	“The presentations were engaging, relatable, and emotionally charged, making them memorable.”
	Emotional engagement	Focus on emotional care was a key strength	“I appreciated the focus on emotions and how they are central to patient care.”
	Interaction with professionals	Valued interactions with diverse experts	“It was valuable to learn from a diverse set of professionals, each with unique insights.”

Regarding workshop improvement, several learners suggested more interactive peer-learning opportunities throughout training, with a preference to include other shorter, more focused sessions. Other requests included additional case-based studies for application of concepts in practice. When reflecting on the best aspects of the workshop, participants praised the engaging presentations and honest emotional focus with one noting, “*The presentations were engaging, reliable and emotionally charged*.” Moreover, the interaction with professionals from other disciplines was highly valued, as participants appreciate the opportunity to learn from other trainees, but also other clinical faculty.

## Discussion

Our innovative curriculum cross-match synergizes subspecialty palliative IPE learning experiences for learners. The primary findings of our study support our initial goal of creating an effective and engaging learning experience, as demonstrated by the significant increase in comfort levels across all interprofessional competency domains. The use of a case-based, interdisciplinary approach to PC education was particularly impactful, with improvements of up to 50% in some areas. The success of active and collaborative learning techniques in achieving these outcomes highlights the value of constructivist and constructionist learning theories in medical education. Furthermore, interdisciplinary learning experiences contributed to learners’ appreciation of interprofessional collaboration, including a deeper understanding of the roles, skills, and perspectives of other specialties in providing comprehensive patient care. Notably, 96% of learners agreed that working with peers from other disciplines enhanced their education, suggesting that early collaboration can improve communication and team-based care in clinical practice.

The findings of our study align closely with existing literature that underscores the significance of case-based, interdisciplinary learning in enhancing communication skills and promoting patient-centered care. A recent systematic review by Kirkpatrick et al. highlights that IPE training not only bolsters self-reported confidence and self-efficacy but also cultivates positive attitudes, clarifies role understanding, and facilitates collaborative practice.^8,26–28^ Furthermore, objective evaluations demonstrate a marked improvement in both PC knowledge and the efficacy of interprofessional PC delivery. This convergence with prior research reinforces the critical role of IPE in advancing quality care in palliative settings.^5,29^

Our team built on existing literature by employing a unique, systematic approach to the needs assessment that informed the development of the most appropriate learning objectives.^11^ Using this structured framework, we defined objectives based on skills, knowledge, and attitudes, resulting in a comprehensive, multidimensional learning experience. The inclusion of learning theories and an instructional design specialist further supported the integration of collaborative learning experiences.

An additional contribution of our study to the literature is the novel grouping of learners. Our cohorts included psychologists alongside physicians and nurses, an interdisciplinary mix not commonly found in traditional PC education. Prior to this workshop, these learners may have encountered each other at weekly interdisciplinary meetings in inpatient PC units but rarely had the opportunity to learn and practice together clinically. Although not captured in the initial dataset, we observed that, following the workshop, learners actively seek each other out for patient support and engage in more dialog during clinical care.

Throughout the project, our team navigated logistical challenges, including faculty turnover and resource support of the workshop. Despite these aspects and with the support from the IPEX team, we effectively managed time constraints for clinicians and educators. To improve sustainability and efficiency, we strategically incorporated a “train-the-trainer” model into our team by involving both senior and junior faculty members from each represented specialty, which also fostered the professional development of junior faculty in IPE to enhance workshop sustainability.^30^ A limitation to the generalizability of our findings is the unique composition of the subspecialty learner group based on our institutional HAPC training programs.

This collaborative IPEX Death and Dying workshop model has the potential for broader implementation across diverse health care settings, advancing interprofessional collaboration in PC. Future efforts should focus on identifying strategies to ensure the sustainability of the workshop, expanding the curriculum to cover key areas such as ethics in PC, incorporating simulation-based learning to improve educational outcomes, and enhancing interdisciplinary collaboration by engaging additional specialties, including social work and chaplaincy.

## Abbreviations Used

### Abbreviations Used

ADDIE: Analysis, Design, Development, Implementation, Evaluation Model
CBL: Case Based Learning
HAPC: Hospice and Palliative Care
IPE: Interprofessional Education
IPEC: Interprofessional Education Collaborative Competencies
IPEX: Interprofessional Education Exchange
NCP: National Consensus Project
PC: Palliative Care
SPICE-R2: Student Perceptions of Physician-Pharmacist Interprofessional Clinical Education Instrument, Version 2
